# Nondestructive Detection of Foreign Matter in Pu-erh Ripe Tea Based on Deep Learning

**DOI:** 10.3390/foods15122083

**Published:** 2026-06-08

**Authors:** Baijuan Wang, Xiaoxue Guo, Xin Fang, He Ji, Jihong Zhou, Junjie He, Shihao Zhang, Yuefei Wang

**Affiliations:** 1 College of Tea Science, Yunnan Agricultural University, Kunming 650201, China; wangbaijuan2023@163.com (B.W.); 18488871225@163.com (X.G.); 15623093593@163.com (X.F.); jihe_ynau_edu@163.com (H.J.); 18087827443@163.com (J.H.); 2 College of Agricultural & Biotechnology, Zhejiang University, Hangzhou 310013, China; zhoujihong@zju.edu.cn; 3 Mechanical & Electrical Engineering College, Wuhan Donghu University, Wuhan 430071, China; 4 Tea Research Institute, Zhejiang University, Hangzhou 310013, China

**Keywords:** tea, visual mechanism, convolution, foreign matter detection, food safety

## Abstract

To address the challenges of small foreign matter size, severe occlusion, and complex backgrounds in Pu-erh ripe tea processing, this study drew inspiration from primate visual mechanisms and proposed an improved YOLOv13-based network, AE-YOLOv13-S. To mitigate loss of fine details, the weakening of discriminative features, and the frequent occurrence of missed and false detections, the Adaptive Sparse Self-Attention Network was introduced to optimize the backbone of the network, inspired by the sequential cognitive pattern of primates involving target search, local verification, selective integration, and final decision making. To address insufficient long-range semantic associations and the submergence of fine-grained differences in background noise, Emulating Self-Attention with Convolution was employed to optimize part of the Conv modules of the network, drawing on the hierarchical information processing mechanisms of primates from peripheral perception to central fine analysis. In response to the limitations of bounding boxes, such as approximate target enclosure, the large amount of geometric supervision noise, the obvious localization deviation, and delayed model convergence, a Scale-based Dynamic Loss, inspired by primate visual perception mechanisms, was introduced to optimize the network’s loss function. The results showed that, during training, compared with the baseline, AE-YOLOv13-S achieved lower training loss values: Box Loss declined by 6.76%, Cls Loss by 6.52%, and DFL Loss by 8.65%. On the validation dataset, the model demonstrated reductions of 6.58%, 16.39%, and 8.33% for these respective metrics. After the overall improvements, AE-YOLOv13-S achieved increases of 1.43, 4.85, and 2.69 percentage points in precision, recall, and mAP@50, respectively, with only a 0.3 G increase in FLOPs. The improved model can classify and detect foreign matter in Pu-erh ripe tea efficiently and accurately, providing not only a new technical pathway for foreign matter detection in tea processing but also a practically meaningful technical solution for intelligent quality control and food safety assurance in the tea processing chain.

## 1. Introduction

As a cultural symbol and healthy beverage that has been passed down for thousands of years in the Chinese nation, tea is rich in functional components such as tea polyphenols and theanine, and it offers multiple health benefits, including mental refreshment, antioxidant activity, and metabolic regulation. Tea itself is also one of the pillar industries promoting rural revitalization in China [1]. According to the China Tea Production and Marketing Situation Report, in 2023, the total area of tea plantations in China reached 51.4976 million mu, of which 46.5016 million mu had been harvested, and tea products were exported to 130 countries and regions. Among all tea categories, dark tea, to which Pu-erh tea belongs, recorded a total output of 458,000 tons, accounting for 13.7% of the national tea output.

With the continuous increase in tea production, intelligent monitoring throughout the entire process from production to consumption has become one of the key measures for ensuring the hygiene and safety of tea products. Among these measures, foreign matter detection, as a crucial step in maintaining tea purity, is especially important for Pu-erh tea that requires a sun-drying process [2]. Exogenous foreign objects, such as bamboo leaf fragments, small pebbles, and small twigs, may be introduced into Pu-erh tea during raw material harvesting, spreading, drying, pile fermentation, transportation, and refined processing. Although these foreign objects are not chemical hazards, they can affect product cleanliness, sensory quality, and consumer acceptance. In particular, hard foreign objects such as small pebbles and small twigs may also pose physical food hazards. Traditional methods for foreign matter detection in Pu-erh tea, including manual sorting, air separation, magnetic separation, and water washing, are inefficient, easily affected by human factors, and incapable of effectively identifying small foreign matter. Moreover, these methods may damage the natural flavor of the tea and therefore cannot meet the demands of large-scale and standardized production in modern agriculture [3]. Consequently, how to achieve rapid and accurate nondestructive detection and localization of foreign matter in Pu-erh tea has become one of the key challenges in preserving its natural flavor and ensuring food safety.

With the maturation and widespread adoption of Artificial Intelligence (AI), computer vision, and image processing technologies in food science, deep learning has been widely applied to the detection and localization of foreign matter in food production [4]. Deep learning–based object detection algorithms can extract key features from large volumes of foreign matter data, thereby enabling accurate detection. Moreover, the integration of bio-inspired vision and deep learning has provided a new technical pathway for the nondestructive detection of foreign matter in Pu-erh tea [5].

To address impurities that may be introduced during the picking and processing of premium green tea, Zezhong Ding et al. adopted YOLOv8 as the base network and optimized it by replacing the loss function, improving lightweight convolution, applying model pruning, and incorporating knowledge distillation [6]. The results showed that the optimized YOLOv8 network achieved 4.2 giga floating point operations, a parameter size of 791,966 bytes, and precision, recall, and mean average precision values of 93.79%, 89.59%, and 94.84%, respectively. Compared with the original YOLOv8 network, giga floating point operations and parameter size were reduced by 48.15% and 73.66%, respectively, while precision, recall, and mean average precision increased by 2.16%, 3.2%, and 2.61%, respectively. The model trained with the improved network maintained relatively high detection accuracy while reducing computational resource consumption to some extent, thereby providing technical support for tea impurity detection.

To address the low detection accuracy of tiny objects under complex background conditions, Sihan Huang et al. proposed a bio-inspired remote sensing tiny object detection model based on the human visual system [7]. By introducing XYW Conv and the XYWA Attention mechanism, the model simulated the parallel processing pathways of X, Y, and W cells in the human visual system, thereby enhancing the contrast between tiny objects and the background, and effectively highlighting key features. To suppress the influence of larger objects in shallow feature maps, Top Down Suppression Attention was further introduced to preserve the features of tiny objects. The results showed that the improved model contained only 1.8 million parameters, achieved an average precision of 10.4% on the AI TOD dataset, and reached 143.5 frames per second, significantly outperforming the models trained with YOLOv8 m and ORFENet. This study not only provided a detection model with low computational cost for tiny object detection, but also demonstrated the application potential of biological vision principles in machine vision tasks.

To address the problems of small foreign matter size, severe occlusion, and complex backgrounds during the processing of Pu-erh ripe tea, this study proposes an efficient detection method based on machine vision [8]. Built upon the YOLOv8 network, the method introduces Receptive Field Attention Convolution, Double Attention Networks, and Shape IoU to enhance the feature extraction capabilities of the baseline network. The results show that the optimized YOLOv8 network achieves precision, recall, and mean Average Precision (mAP) values of 96.76%, 96.74%, and 98.12%, respectively, which correspond to improvements of 3.73%, 5.18%, and 3.59% compared with the original network values of 93.03%, 91.56%, and 94.53%. This study not only provides an efficient and intelligent tool for foreign matter detection in Pu-erh ripe tea, but also establishes a foundation for the intelligent control of food safety.

Existing studies indicate that deep learning, attention mechanisms, and bio-inspired approaches demonstrate significant technical potential and methodological value for the nondestructive detection of agricultural products, providing a theoretical basis and technical reference for online foreign object recognition during tea processing [9]. However, for the complex scenario of foreign object detection in Pu-erh ripe tea, existing approaches still struggle to satisfy practical requirements. Under complex background conditions, current algorithms exhibit insufficient discriminative representation of fine-grained differences between foreign objects and tea leaves; they also have limitations in modeling long-range dependencies across spatial regions. Although some bio-inspired vision studies have shown considerable innovation in simulating cognitive mechanisms, their methodological frameworks are mainly designed for specific application scenarios, such as remote sensing. They have not yet developed a well-suited optimization framework to address the compound challenges in tea foreign object detection, including weakly salient targets, complex natural backgrounds, and visually similar interference.

In response to the challenges outlined above, this study was grounded in the practical need for nondestructive foreign matter detection in Pu-erh ripe tea and focused on several key bottlenecks, including complex background interference, local occlusion, weak saliency of small targets, and limited localization accuracy [10]. Based on the YOLOv13 network [11], the study drew on the sequential cognitive characteristics of primates in target search, local verification, selective integration, and final decision making. It then introduced the Adaptive Sparse Self-Attention Network to enhance the network’s representational capability [12]. The local spatially adaptive feature estimation mechanism is employed to improve the model’s perceptual sensitivity to texture variations and fine-grained abnormal patterns. The sparse self-attention mechanism is used to selectively filter candidate responses and suppress redundant background responses and irrelevant interference. By combining the gated feed-forward module with the residual fusion strategy, key information is enhanced, selectively filtered, and integrated collaboratively, thereby constructing more discriminative feature representations.

Drawing on the hierarchical information processing mechanism of primates from peripheral perception to central fine analysis, Emulating Self-Attention with Convolution was adopted to optimize the network structure [13]. While preserving the advantage of Transformer in global dependency modeling as much as possible, this approach effectively reduced the computational complexity and storage cost caused by the stacking of multiple self-attention layers. By using convolution operators to approximately simulate the long-range dependency modeling capability and adaptive weighting mechanism of self-attention, the model achieved improved representational efficiency in complex scenarios.

Inspired by the scale-adaptive regulation mechanism in biological visual perception, Scale-based Dynamic Loss was employed to enhance the object regression process [14]. According to differences in target scale, the supervision weights of the position term and the scale term were dynamically reassigned. For small targets, the intensity of position supervision was enhanced, while the excessive sensitivity of IoU based geometric overlap measures to slight offsets was moderately reduced. For large targets, conventional geometric overlap constraints were retained, thereby enabling adaptive regression optimization for targets of different scales and improving the stability and accuracy of bounding box localization. This study aimed to construct a highly robust foreign matter detection model for the complex processing scenario of Pu-erh ripe tea through the collaborative optimization of the YOLOv13 network, so as to achieve accurate recognition and stable localization of foreign matter targets under complex backgrounds, local occlusion, and weak saliency of small-scale targets. It also seeks to provide a theoretical basis and technical support for intelligent quality control and food safety assurance in tea processing, while promoting bio-inspired computing strategies in complex visual perception and intelligent detection.

## 2. Materials and Methods

### 2.1. Dataset Construction

To ensure diversity in the experimental data, this study selected Pu-er City and Xishuangbanna Prefecture in Yunnan Province, located at 22.81° N, 100.96° E and 21.45° N, 100.28° E, respectively, as the data collection regions. As the origin of the Ancient Tea Horse Road, Pu-er City has an elevation ranging from 376 m to 3306 m, a forest coverage rate exceeding 67%, and a total tea plantation area of 2.177 million mu, including 0.19 million mu of ancient tea gardens and 1.987 million mu of modern tea gardens. Xishuangbanna Prefecture is humid and rainy year-round, with a forest coverage rate of 59.26% and a total tea plantation area of 1.4314 million mu, which accounts for 19.25% of Yunnan Province’s total tea plantation area. As major tea-producing regions in Yunnan, Pu-er City and Xishuangbanna Prefecture are highly representative in terms of ecogeographical characteristics. Moreover, the marked differences in climate and ecosystem structure between these regions provide a natural advantage for enhancing the generalization capability of the developed model [15].

Image data were acquired using a Canon EOS R5 camera (Canon China Co., Ltd., Beijing, China) equipped with an RF 24–105 mm (Canon China Co., Ltd., Beijing, China) lens to ensure high-quality sample data in terms of spatial resolution, texture representation, and detail fidelity. The distance between the lens and the Pu-erh ripe tea samples was set to 10–20 cm to simulate the distance between sorting equipment and tea materials in actual production. To suppress image noise and enhance target details, the ISO sensitivity was uniformly set to 200. An aperture of f/4.0 was used to balance depth of field and exposure, thereby improving the distinguishability of surface textures, edge contours, and local features of both tea and foreign objects. The shutter speed was set to 1/300 s to avoid motion blur. Acquired images had an original resolution of 5152 × 3864 pixels and were saved in JPG format. Illumination was provided by a 24-inch flat-panel fill light with an input voltage of 220 V and a color temperature of 5500 K. A white background was used to simulate a conveyor belt scenario. The camera was positioned vertically, and tea and foreign-object samples were randomly arranged within the background area to improve the realism of spatial distribution. Tea samples were kept stationary during image acquisition to ensure annotation accuracy [16].

In total, 543 images of foreign objects in Pu-erh ripe tea were collected, with each image corresponding to a single sample. The sample types included three categories: bamboo leaf fragments, small twigs, and small pebbles. During preprocessing, images were evaluated and screened for clarity, validity, and sample independence. After removing blurred images, duplicate samples, and invalid data, 512 high-quality images were retained to construct the dataset. A total of 983 foreign object instances were annotated, including 347 bamboo leaf fragments, 364 small pebbles, and 272 small twigs. Annotation was jointly completed by three associate professors to ensure credibility and professionalism. Subsequently, annotation results were reviewed and consistently corrected for target boundaries, category assignments, and occluded regions in cases of disagreement. Finally, to balance the sample requirements for model training with the objectivity of experimental evaluation, the dataset was partitioned into training, validation, and test subsets at a ratio of 6:2:2 [17].

### 2.2. Data Augmentation

**Figure 1:**
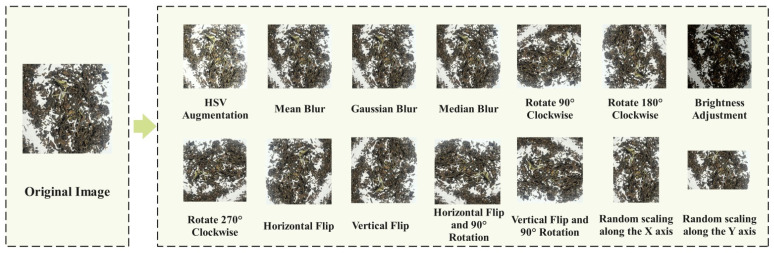
Data augmentation.

To improve the model’s generalization ability, environmental adaptability, and overall recognition accuracy for detecting foreign matter in Pu-erh ripe tea, several data augmentation strategies were incorporated into the training pipeline, as illustrated in Figure 1. To effectively simulate the color variations caused by different lighting conditions, color temperature shifts, and imaging device discrepancies, HSV Augmentation was applied. This method imposes random perturbations on the hue, saturation, and value channels, thereby enhancing the model’s representational stability and robustness in complex color environments [18]. Specifically, the hue component was randomly shifted within a range of ±5.4 degrees. The saturation and value components were multiplied by coefficients randomly sampled from [0.3, 1.7] and [0.2, 1.8], respectively. This wide range, particularly for the value channel, was chosen to simulate extreme low-light and high-exposure scenarios. To construct diverse scenarios of image quality disturbance, Mean Blur, Gaussian Blur, and Median Blur were introduced with a fixed kernel size of 7 × 7 to simulate image clarity degradation [19]. The kernel size for mean blur, Gaussian blur, and median blur was set to 7 × 7. Among them, Mean Blur was mainly used to simulate variations in visual features under conditions of reduced image resolution or compression distortion, with its core mechanism lying in the weakening of local texture details through the replacement of neighborhood pixels by their mean values. Gaussian Blur smoothed the image on the basis of a Gaussian kernel function and could more realistically characterize blur phenomena caused by imaging jitter, slight defocus, or dynamic acquisition processes, thereby enhancing the feature representation capability of the model. Median Blur was mainly used to suppress impulse noise such as salt and pepper noise, and it preserved edge contours and structural information while effectively removing noise, thus improving the model accuracy in recognizing target edge features and morphological structures under complex backgrounds. In terms of lighting enhancement, Brightness Adjustment was used to randomly modify the overall image brightness so as to simulate imaging differences under different time periods and varying environmental illumination intensities, thereby enhancing the robustness and generalization ability of the model under nonuniform illumination and lighting fluctuation conditions [20]. For geometric augmentation, a combination of D4 transformation and random anisotropic scaling was implemented. The D4 transformation expanded the pose distribution of samples through discrete square-angle rotations (0°, 90°, 180°, 270°) and flips, effectively simulating the different arrangement directions of foreign objects during sorting. Furthermore, random anisotropic scaling, with horizontal and vertical ratios independently sampled from [0.5, 1.5], was applied to increase the diversity of detected objects in terms of scale, morphology, and viewing perspective, thus improving the model’s adaptability to these variations [21].

**Figure 2:**
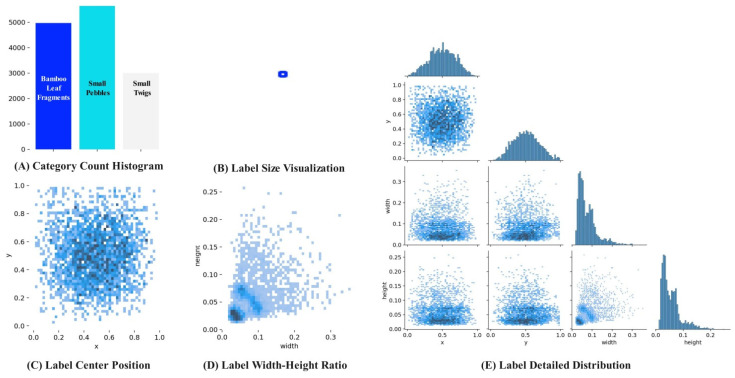
Sample distribution of the training set after data augmentation. (**A**) Category count histogram. (**B**) Label size visualization. (**C**) Label center position. (**D**) Label width-height ratio. (**E**) Label detailed distribution. A darker color indicates a higher density of data points.

Figure 2 shows the sample distribution of the enhanced and filtered training sets. Figure 2A shows the histogram of the foreign matter category of Pu-erh ripe tea. Figure 2B illustrates the scale distribution of annotated targets in terms of the width–height ratios, with bounding box center points normalized and mapped to the image center. Figure 2C displays the spatial distribution of foreign matter within the image plane. Figure 2D is used to characterize the morphological scale features of the targets. Figure 2E further presents the fine-grained feature information of the labels in the images. Here, X and Y denote the position of the object, while width and height represent its dimensions. A darker color indicates a higher density of data points.

### 2.3. Improvement of the YOLOv13 Network

Compared with traditional detection frameworks, YOLO has demonstrated strong application advantages in complex industrial vision inspection scenarios because of its technical characteristics, such as single-stage design, end-to-end processing, and high real-time performance [22]. As a new generation network in the YOLO series released in 2025, the core of YOLOv13 lies in the introduction of a hypergraph-based adaptive correlation enhancement mechanism and an aggregation-to-distribution feature organization paradigm throughout the detection process [23]. This design effectively strengthens feature interaction across spatial positions and scale levels, thereby achieving a coordinated improvement in detection accuracy and inference efficiency under the constraints of a lightweight architectural design. This characteristic shows a high degree of compatibility with the practical requirements of nondestructive foreign matter detection in Pu-erh ripe tea in terms of efficiency, accuracy, and deployment feasibility. Compared with currently widely used networks such as YOLOv10, YOLOv13 offers a stronger structural basis for feature interaction, multi-scale information fusion, and lightweight detection. However, when foreign matter targets exhibit complex phenotypic characteristics such as small scale, weak texture, low saliency, or local occlusion, the effective representation of these targets may still be limited due to insufficient spatial information. In particular, under the combined effects of complex backgrounds, strong texture interference, and dense multiscale distributions, the model still suffers from missed detections, false detections, and localization deviations.

**Figure 3:**
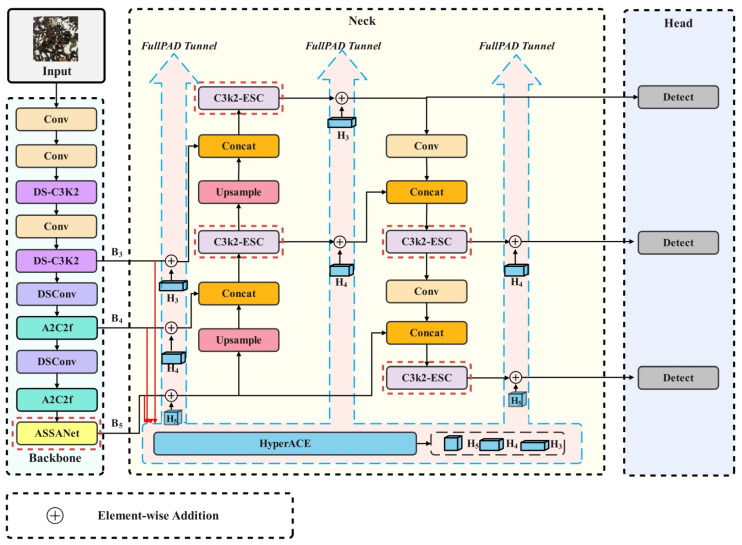
Improved YOLOv13 network.

To address these challenges, this study draws on the biologically inspired sequential cognitive characteristics of primates, including target search, local verification, selective integration, and final decision making. Based on this concept, the Adaptive Sparse Self-Attention Network was adopted to optimize the backbone of the network. To enhance discriminability, improve long-range semantic associations, and prevent the submergence of fine-grained differences in background noise caused by dense tea leaf stacking, interlaced edges, and heterogeneous scale distribution, Emulating Self-Attention with Convolution was introduced to optimize part of the convolutional modules, drawing on the hierarchical information processing mechanisms of primates from peripheral perception to central fine analysis. To overcome limitations of bounding boxes, increased geometric supervision noise, localization deviations, and delayed convergence caused by small-scale, discrete, irregularly shaped, and blurred foreign objects, Scale-based Dynamic Loss was adopted to optimize the network’s loss function, inspired by biologically inspired visual perception mechanisms. The overall structure of the improved network is shown in Figure 3, and the detailed parameters are presented in Table 1. The red dotted box represents the optimized module. B3, B4 and B5 represent the multi-scale feature maps output from Backbone. H3, H4 and H5 represent the enhanced feature maps after HyperACE treatment. DS-C3k2 represents a lightweight feature extraction module based on C3k2. DSConv represents deep separable convolution for lightweight feature extraction. A2C2f represents the cross-stage part of regional attention with fast feature extraction. HyperACE is a hypergraph-based adaptive correlation enhancement, which is mainly used for high-order correlation modeling and feature enhancement of multi-scale features across different scales and spatial locations. Full PAD _ Tunnel is Full Pipeline Aggregation and Distribution Tunnel, and its core idea is to distribute the enhanced features to different parts of the network. We use DownsampleConv to reduce the spatial resolution of the feature map and adjust its feature dimension.

#### 2.3.1. Optimization with the Adaptive Sparse Self-Attention Network

When detecting foreign matter in Pu-erh ripe tea, the visual appearance of tea leaves under natural spreading and withering conditions exhibits significant heterogeneity. Factors such as uneven color, leaf curling, and local occlusion often result in pronounced texture interference in the images. Meanwhile, foreign matter targets typically present small-scale, large morphological variation, and local occlusion, further increasing the difficulty of feature representation. Although the YOLOv13 network can effectively extract global semantic features, its feature extraction process still suffers from limitations such as the loss of detailed information and the weakening of discriminative features when dealing with foreign matter targets that rely on fine high-frequency texture details and local morphological variations. In particular, in complex scenarios where foreign matter coexists with pseudo foreign matter such as scorched leaf edges, the model is more easily affected by redundant local background responses and visual confusion, resulting in frequent missed detections and false detections.

**Figure 4:**
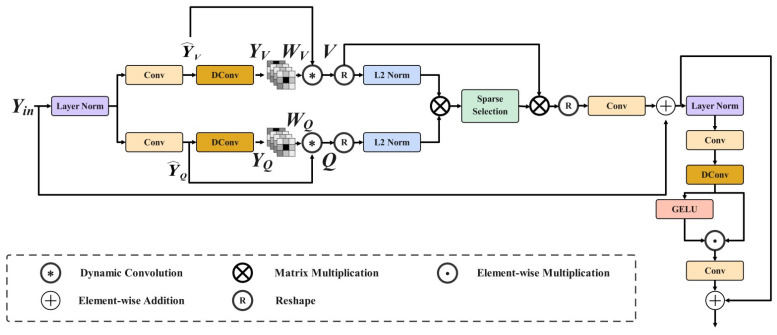
Adaptive Sparse Self-Attention Block.

Inspired by the sequential cognitive pattern of primates under complex background conditions—which includes target search, local verification, selective integration, and final decision making [24]. Through the rapid capture of local structural changes, texture anomalies, and morphological differences, the visual system initially generates potential target candidate regions. With the aid of a selective attention mechanism, limited perceptual and computational resources are concentrated on a small number of key cues with high discriminative value. It then integrates contextual semantic relationships within a larger receptive field to achieve fine-grained recognition and reliable judgment of abnormal targets. Inspired by this visual cognitive mechanism, this study introduced the Adaptive Sparse Self-Attention Network into the YOLOv13 network for optimization, as shown in Figure 4. The core idea lies in enhancing network responsiveness to local structural variations and subtle abnormal cues through locally adaptive spatial feature estimation. On this basis, a sparse self-attention mechanism was used to adaptively select and filter a large number of candidate responses, retain a small amount of long-range dependency information with high discriminative contribution, and suppress redundant responses and noise interference. Finally, key information was further enhanced, suppressed, and collaboratively integrated through a gated feedforward module and a residual fusion mechanism, thereby gradually forming a more robust discriminative representation for abnormal target identification.

The input feature $Y_{in}$ generates dynamic kernels $W_{V}$ and $W_{Q}$ through Layer Norm and convolution operations, where ${\hat{Y}}_{V}$ denotes the initial projected feature of the value branch, ${\hat{Y}}_{Q}$ denotes the initial projected feature of the query branch, and $Y_{V}$ and $Y_{Q}$ denote the local features after depthwise convolution. Dconv (Dynamic Convolution) is then used to generate *Q* and *V* with locally adaptive spatial variability, which represent the final query and value features, respectively. For the output features, a sparse selection mechanism is further adopted to form the attention matrix A, which retains only strongly correlated dependencies, as shown in Equation (1). Here, $\hat{Q}$ and $\hat{V}$ denote the reshaped query and value matrices, γ denotes the scaling factor, and S denotes the selection function. Sparse attention aggregation and residual updating are shown in Equations (2) to (3), where T denotes the reshape operation used to restore the matrix form result to the feature map form. ${\hat{X}}^{g}$ denotes the intermediate feature after attention aggregation. The channel dimension is then expanded, and depthwise convolution is used to mix local information. Finally, the feature is divided into two branches for gated multiplication and then compressed back to the original channel dimension.

(1) $$A=S\left({\gamma \hat{Q}\hat{V}}^{⊤}\right),\;A\;\epsilon \;R^{C\times C}$$

(2) $${\hat{X}}^{g}=T\left(AV\right)$$

(3) $$X^{g}={Conv}_{1\times 1}\left({\hat{X}}^{g}\right)+Y_{i}$$

#### 2.3.2. Optimization with Emulating Self-Attention with Convolution

In the task of foreign matter detection in Pu-erh ripe tea, the structural characteristics of tea leaves, including high density stacking, interlaced edges, and heterogeneous scale distribution, create a highly complex and irregular visual background. Consequently, target regions often exhibit pronounced texture redundancy, morphological occlusion, and boundary uncertainty. In addition, multiple types of foreign matter show high visual similarity to the tea itself in terms of color representation and brightness response, which significantly reduces the discriminability between targets and the background. Existing object detection methods mainly rely on hierarchical feature extraction mechanisms driven by local receptive fields. While these methods efficiently encode neighborhood patterns, they face clear limitations in modeling long-range dependencies, capturing cross-region semantic associations, and preserving fine-grained difference representations. In complex scenarios with high background similarity, the model struggles to distinguish subtle visual differences between foreign matter and tea leaves, causing key discriminative cues to be submerged in background noise, leading to feature representation shifts, degradation of class decision boundaries, and increased false and missed detections.

**Figure 5:**
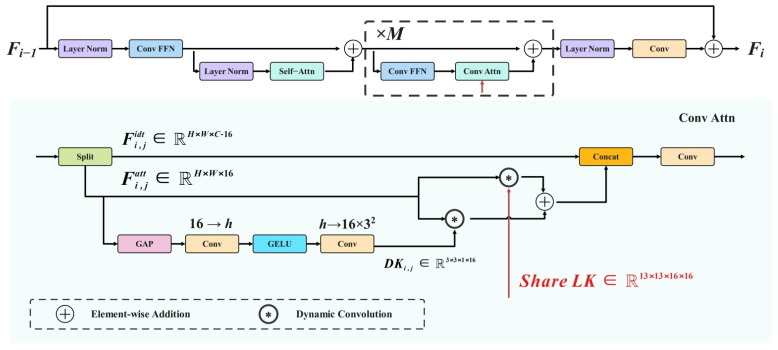
Emulating Self-Attention with Convolution.

During visual recognition, primates usually adopt a hierarchical information processing mechanism that progresses from peripheral perception to central fine analysis [25]. Peripheral vision provides a rapid preattentive scan of the scene over a large field of view, prioritizing global cues such as spatial layout, motion variation, and saliency distribution. This enables preliminary localization of potential targets. Under the regulation of the selective attention mechanism, limited visual resources are focused on candidate target regions, and the targets are mapped to the foveal region through saccadic eye movements. Because the fovea has the highest spatial resolution, the visual system can further perform fine encoding and high-confidence discrimination of target shape features, texture patterns, boundary information, and local details. Inspired by the operational mechanism of this biological pattern, this study adopted Emulating Self-Attention with Convolution to perform targeted optimization of the YOLOv13 network, as shown in Figure 5.

The core idea of Emulating Self-Attention with Convolution is to preserve the global representation advantage of the Transformer as much as possible while significantly reducing the memory access burden caused by repeated stacking of self-attention layers. For the input feature $F_{i-1}$, local spatial information is first adaptively encoded and enhanced through Layer Norm and the convolutional feedforward network, thereby producing the locally enhanced feature, as shown in Equation (4). Here, LN denotes Layer Norm, ConvFFN denotes the convolutional feedforward network, and $F_{i}^{in}$ denotes the locally enhanced feature. On this basis, in order to reduce computational redundancy, the network retains only a single window self-attention operation and generates $F_{i,0}$ through residual connection, as shown in Equation (5). Unlike the conventional Transformer design that repeatedly stacks self-attention layers within each block, Emulating Self-Attention with Convolution replaces multilayer attention stacking in the subsequent stage with M residual substructures composed of ConvFFN and ConvAttn. In this way, convolution is used to emulate the long-range dependency modeling capability and input adaptive weighting ability of self-attention, as shown in Equation (6), where LK denotes the shared large kernel. Finally, the network completes feature fusion through Layer Norm and convolution operations and, under the residual connection mechanism, adds the fused feature to the initial input to generate the final output feature $F_{i}$.

(4) $$F_{i}^{in}=ConvFFN(LN(F_{i-1}))$$

(5) $$F_{i,0}=F_{i}^{in}+SelfAttn(LN(F_{i}^{in}))$$

(6) $$F_{i,j}=F_{i,j-1}+{ConvAttn}_{j}\left({ConvFFN}_{j}\left(F_{i,j-1}\right),LK\right),\;j=1,\ldots ,M$$

#### 2.3.3. Optimization with Scale-Based Dynamic Loss

In foreign matter detection for Pu-erh ripe tea, stable and clearly defined high-contrast boundaries are often absent between foreign matter and tea leaves. Furthermore, foreign matter regions are easily affected by coupled interference from tea trichomes, vein textures, fractured edges, and illumination shadows. This results in considerable uncertainty and ambiguity in the contour representation of targets. Although YOLOv13 can efficiently extract global semantic features through its backbone and feature fusion architecture, its detection head still relies on a bounding-box regression paradigm. Given that foreign matter typically exhibits small-scale, discrete distribution, irregular morphology, and blurred edges, annotated boxes often provide only approximate enclosures of the targets. Consequently, the network during training is more likely to be driven by noisy supervision rather than by effective geometric cues.

By contrast, in biological visual perception, when primates are in complex environments characterized by vegetation occlusion, insufficient illumination, or strong target camouflage, their perceptual process does not usually rely on precise recognition of target shape at the initial stage. Instead, they first use relatively stable environmental cues, such as motion disturbance, spatial position changes, abnormal local contrast, and olfactory signals, to conduct coarse screening and rapid localization of potential target regions. After a preliminary judgment of target presence has been formed, attentional resources are gradually concentrated on the candidate regions. By narrowing the perceptual range, the visual system further integrates higher-level features, including contour, morphology, and detail, so as to achieve fine-grained target recognition and form the corresponding attack decision [26].

To improve target localization accuracy and accelerate model convergence in foreign matter detection, this study introduced Scale-based Dynamic Loss to reconstruct the loss function system of YOLOv13. The core idea is to dynamically adjust the optimization focus of regression supervision according to target scale. For small-scale targets, positional supervision is emphasized to mitigate the excessive sensitivity of geometric overlap measures to slight offsets. For large-scale targets, the conventional regression optimization dominated by geometric overlap constraints is retained. This enables adaptive allocation and coordinated integration of supervision mechanisms for targets of different scales.

The bounding box regression error, under the optimized Scale-based Dynamic Loss, is split into scale and position components, as specified by Equations (7) and (8). Here, $L_{BS}$ denotes the scale loss, and $L_{BL}$ denotes the position loss. IoU denotes the intersection over union between the predicted box and the ground truth box, αν denotes the aspect ratio consistency term of the bounding box, ρ represents the Euclidean distance between the predicted center point $b_{p}$ and the ground truth center point $b_{gt}$, while c refers to the diagonal length of the smallest enclosing box covering both predictions. The scale term is used to characterize the consistency between the predicted box and the ground truth box in terms of overlap area and shape ratio, whereas the position term is used to characterize the relative offset between the center of the predicted box and the center of the ground truth box.

(7) $$L_{BS}=1-IoU+\alpha \nu$$

(8) $$L_{BL}=\frac{\rho^{2}(b_{p},b_{gt})}{c^{2}}$$

On this basis, scale-based dynamic loss no longer uses fixed weighting. Instead, it generates a dynamic coefficient $\beta_{B}$ according to the true scale of the target and further constructs complementary weights for the scale term and the position term, as shown in Equation (9). Here, $R_{OC}$ denotes the scale recovery ratio, whose core function is to map the target area on the current layer back to a scale reference system consistent with that of the original image. $w_{o}$ and $h_{o}$ denote the width and height of the original image, whereas $w_{c}$ and $h_{c}$ denote the current feature map width and height. $B_{gt}$ denotes the area of the current ground truth bounding box, and δ denotes an adjustable hyperparameter used to control the variation range of the dynamic weight.

(9) $$\beta_{B}=min(\frac{B_{gt}}{B_{gt\;max}}\times R_{OC}\times \delta ,\delta )$$

(10) $$R_{OC}=\frac{w_{o}\times h_{o}}{w_{c}\times h_{c}}$$

The final scale-based dynamic loss is shown in Equation (11), where $\beta_{LBS}$ and $\beta_{LBL}$ denote the influence coefficients of the scale and position terms, respectively. In small target scenarios, both $B_{gt}$ and $\beta_{B}$ are relatively small, and scale-based dynamic loss adaptively weakens the contribution of the scale term while increasing the role of the position term. The core rationale is that the spatial extent of a small target box is limited, and even a slight boundary offset can cause a substantial fluctuation in IoU. By comparison, the deviation of the target center point usually shows greater robustness and consistency, and can therefore provide more stable and reliable positional guidance. Shifting the focus of regression optimization to the position term can effectively alleviate unstable gradients caused by annotation errors and ambiguous boundaries.

(11) $$L_{SDB}=\beta_{LBS}\times L_{BS}+\beta_{LBL}\times L_{BL}$$

(12) $$\beta_{LBS}=1-\delta +\beta_{B}$$

(13) $$\beta_{LBL}=1+\delta -\beta_{B}$$

### 2.4. Model Evaluation Metrics

For a systematic assessment of the enhanced YOLOv13 architecture on foreign matter identification in Pu-erh ripe tea, this study established a model evaluation system using precision, recall, F1, AP@50, mAP@50, and mAP@50:95 [27]. Precision, as shown in Equation (14), was mainly used to measure the discriminative accuracy of the model for foreign matter and its ability to suppress false detections. A higher value indicates a stronger ability of the model to distinguish foreign matter from background noise, tea leaf textures, and similar interfering objects. Here, $T_{P}$ denotes the number of targets correctly identified as foreign matter, and $F_{P}$ denotes the number of targets incorrectly classified as foreign matter. Recall, as shown in Equation (15), was used to represent the proportion of actual foreign matter in the samples that was successfully detected by the model. Here, $F_{N}$ denotes the number of foreign matter targets that were not identified by the model. F1, as shown in Equation (16), is the harmonic mean of precision and recall, and it was mainly used to comprehensively characterize the overall balance of the model between false detection control and missed detection control. AP and mAP, as shown in Equations (17) to (18), were also adopted as evaluation metrics. AP@50 refers to the average precision of the model for detecting a single class of foreign matter when the IoU threshold is set to 0.5. mAP@50 denotes the comprehensive evaluation metric obtained by computing the mAP across all object categories at a given IoU threshold, and it was used to reflect the global generalization ability and robust detection performance of the model in multiclass foreign matter detection scenarios. mAP@50:95 represents a comprehensive average evaluation of detection precision under multiple IoU thresholds ranging from 0.50 to 0.95 with an interval of 0.05.

(14) $$Precision=\frac{T_{P}}{T_{P}+F_{P}}\;$$

(15) $$\;\;Recall=\frac{T_{P}}{T_{P}+F_{N}}\;$$

(16) $$F1=2\times \frac{Precision*Recall}{Precision+Recall}$$

(17) $$AP=\sum_{i=1}^{n-1}\left(r_{i+1}-r_{i})P_{inter}(r_{i}+1\right)$$

(18) $$mAP=\frac{\sum_{i=1}^{k}{AP}_{i}}{k}$$

## 3. Results and Analysis

To systematically evaluate the practical performance of the improved AE-YOLOv13-S network in the foreign matter detection task for Pu-erh ripe tea, this study selected seven object detection networks, namely AE-YOLOv13-S, YOLOv13, RT-DETR, SSD, YOLOv10, Faster RCNN, and RTMO, and conducted comparative experiments and performance tests on a unified dataset [28]. In the AE-YOLOv13-S network, A denotes optimization with the Adaptive Sparse Self-Attention Network, E denotes optimization with Emulating Self-Attention with Convolution, and S denotes optimization with Scale-based Dynamic Loss. To guarantee that the findings remain both reproducible and comparable in terms of the experimental results, as well as the reliability of the research conclusions, all networks were trained and tested under the same software and hardware environment, and the detailed experimental settings are presented in Table 2.

To effectively control the interference of confounding variables and ensure the scientific rigor of the model comparison experiments as well as the credibility of result interpretation, this study adopted unified training parameters and optimization settings for all ablation experiment networks, as detailed in Table 3 [29].

In the model comparison experiments, YOLOv13 used the YOLOv13n variant, SSD used the PyTorch 1.3.0 version, YOLOv10 used the YOLOv10n variant, Faster RCNN used version v3.1, and RTMO used the RTMO-S variant. To ensure the objectivity and consistency of the model comparison experiments, all networks were trained using their default training parameters and corresponding pretrained weights. The input image size of all models was uniformly adjusted to 640 × 640 to reduce the influence of input scale differences on the comparison of detection performance and computational complexity.

### 3.1. Comparative Experiment on Data Augmentation

To verify the effect of the data augmentation strategy on improving model generalization ability and detection stability, this study conducted comparative experiments before and after data augmentation using seven object detection networks, namely AE-YOLOv13-S, YOLOv13, RT-DETR, SSD, YOLOv10, Faster RCNN, and RTMO. The experimental results are shown in Table 4. The results show that, after data augmentation was applied, all detection models achieved varying degrees of improvement in Precision, Recall, mAP@50, and mAP@50:95. Overall, the average Precision of the seven models increased by 2.83 percentage points, Recall increased by 2.48 percentage points, mAP@50 increased by 2.63 percentage points, and mAP@50:95 increased by 3.26 percentage points. Among them, after data augmentation, AE-YOLOv13-S achieved Precision, Recall, mAP@50, and mAP@50:95 values of 92.62%, 93.34%, 94.91%, and 85.67%, respectively, representing improvements of 2.36, 2.11, 2.27, and 2.83 percentage points compared with the results without data augmentation. It also maintained the best detection performance among all models. Data augmentation can, to some extent, alleviate insufficient feature learning caused by the limited size of the original samples, significant differences in target scale, and interference from complex backgrounds. It also enhances the robustness of the model to illumination variations, image blur, changes in spatial posture, and scale disturbances, thereby further improving recognition accuracy and localization stability in the foreign object detection task for Pu-erh ripe tea.

### 3.2. Analysis of Model Results

**Figure 6:**
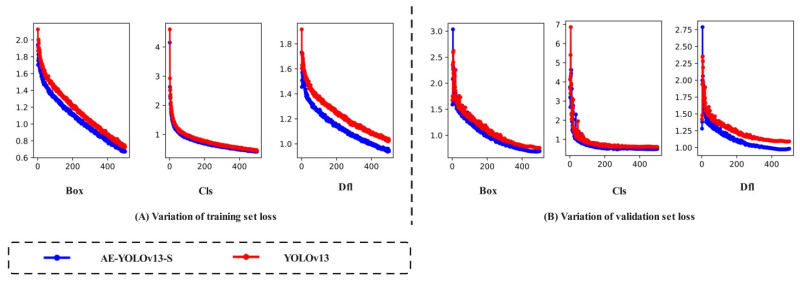
Variation in loss values.

As a core indicator for measuring the degree of deviation between model predictions and ground truth annotations, the loss function serves to transform target localization error, classification bias, and bounding box regression accuracy into quantifiable and optimizable mathematical representations [30]. Through the backpropagation mechanism, it continuously guides network parameters toward globally improved iterative updates, thereby enabling the progressive enhancement of detection performance. In the nondestructive foreign matter detection task for Pu-erh ripe tea, a lower loss value indicates a smaller deviation between the predicted bounding boxes and the ground truth annotations of foreign matter targets. As shown in Figure 6, during the training stage, the Box, Cls, and DFL Loss values of AE-YOLOv13-S dropped below 0.69, 0.43, and 0.95, respectively. Compared with the corresponding values of 0.74, 0.46, and 1.04 for YOLOv13, these losses decreased by 6.76%, 6.52%, and 8.65%, respectively. During the validation stage, the Box Loss, Cls Loss, and DFL Loss of AE-YOLOv13-S remained stable below 0.71, 0.51, and 0.99, respectively, representing reductions of 6.58%, 16.39%, and 8.33% compared with the baseline values of 0.76, 0.61, and 1.08. The Box Loss, Cls Loss, and DFL Loss of the AE YOLOv13 S network on both the training and validation sets were lower than those of YOLOv13, indicating that the improved network can effectively enhance the final model’s ability to learn and represent the features of foreign objects in Pu-erh ripe tea.

**Figure 7:**
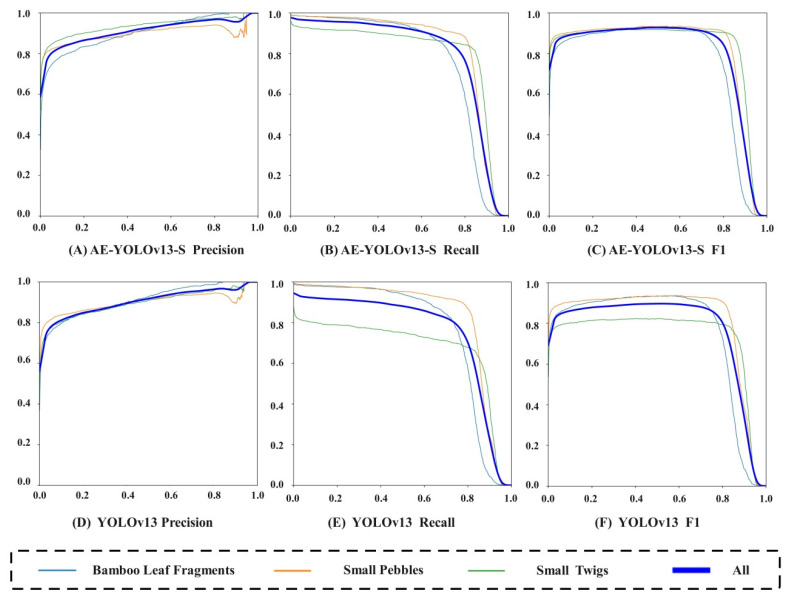
Comparison of precision, recall, and F1.

As shown in Figure 7, AE-YOLOv13-S attained precision, recall, and F1 score values of 92.62%, 93.34%, and 92.98%, respectively. Compared with the corresponding values of 91.19%, 88.49%, and 89.82% for the original YOLOv13, these metrics increased by 1.43, 4.85, and 3.16 percentage points, respectively. The optimized AE-YOLOv13-S demonstrated stronger overall detection capability in the foreign matter detection task for Pu-erh ripe tea. In particular, when dealing with complex scenarios involving small-scale targets, weakly salient targets, and substantial background interference, it effectively improved discriminative feature representation and target response intensity, while significantly enhancing the stability and robustness of foreign matter recognition under complex background conditions.

**Figure 8:**
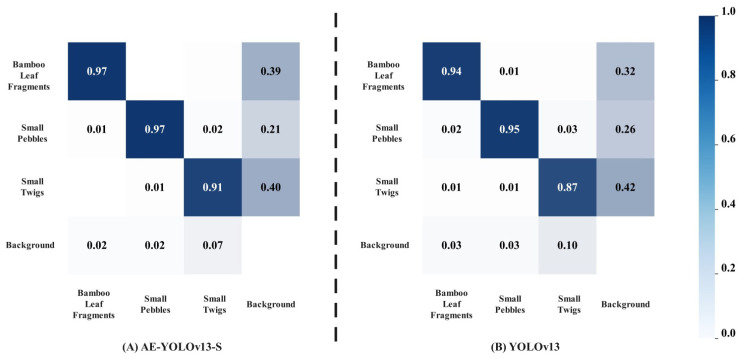
Confusion matrix.

In the study of nondestructive foreign matter detection in Pu-erh ripe tea, the confusion matrix was employed to assess the model’s inter-class discrimination and the sharpness of decision boundaries across foreign matter types [31]. As shown in Figure 8, the rows of the matrix denote the actual foreign matter labels, while the predicted assignments are given by the columns. The values in the diagonal region reflect the degree to which the model correctly identifies each category of foreign matter. A higher value indicates stronger feature representation ability and better category discrimination for that class. The distribution of values in the off diagonal region reflects the extent of misclassification and the pattern of interclass confusion. Compared with the baseline model, AE-YOLOv13-S improved the detection accuracy for bamboo leaf fragments, small pebbles, and small twigs by 3, 2, and 4 percentage points, respectively. Compared with the original YOLOv13, AE YOLOv13 S achieved a 2.69 percentage point improvement in mAP@50. The proposed improvement strategy effectively strengthened the ability of the model to represent discriminative features of different foreign matter categories and enhanced its perceptual sensitivity and category discrimination for fine grained foreign matter under complex background conditions, thereby alleviating missed detections and false detections to a certain extent.

### 3.3. Ablation Study

To verify the effectiveness of AE-YOLOv13-S in the nondestructive foreign matter detection task for Pu-erh ripe tea, and to systematically evaluate the contributions of Adaptive Sparse Self-Attention Network, Emulating Self-attention with Convolution, and Scale-based Dynamic Loss to the performance improvement of YOLOv13, this study conducted ablation experiments on the constructed dataset to quantitatively analyze the gain effects of each improvement strategy on model representation capability, detection accuracy, and overall performance optimization. As shown in Table 5, after optimization using the Adaptive Sparse Self-Attention Network, the Precision, Recall, mAP@50, and mAP@50:95 of the model increased by 0.44, 2.98, 1.16, and 1.44 percentage points, respectively, while the FLOPs increased by only 0.2 G. The sparse self-attention mechanism significantly enhances the model’s ability to represent long-range dependencies and key salient regions with limited additional computational cost, thereby effectively alleviating missed detections caused by the weak saliency and high visual ambiguity in complex tea-leaf backgrounds. After optimization using Emulating Self-Attention with Convolution, the Precision, Recall, mAP@50, and mAP@50:95 of the model increased by 0.39, 2.59, 0.94, and 0.67 percentage points, respectively, while the FLOPs increased by only 0.1 G. This method effectively improves the model’s ability to integrate cross regional contextual relationships and multi scale semantic information while controlling computational complexity, thereby enhancing detection performance in complex scenarios involving small foreign objects, blurred edges, partial occlusion, or low contrast. Optimization using Scale-Based Dynamic Loss improved the model’s Precision by 0.66 percentage points, Recall by 2.16 percentage points, mAP@50 by 0.65 percentage points, and mAP@50:95 by 0.51 percentage points without changing the FLOPs, Parameters, or Gradients of the original model. The loss function design, which dynamically assigns the focus of supervision according to target scale, effectively alleviates the excessive sensitivity of IoU regression to slight boundary shifts in small object scenarios. It also enhances the stability and reliability of localization supervision, thereby improving the model’s localization robustness for fine-grained foreign objects and its training convergence. After the overall improvements, compared with the original YOLOv13, AE YOLOv13 S achieved increases of 1.43, 4.85, 2.69, and 3.02 percentage points in Precision, Recall, mAP@50, and mAP@50:95, respectively. Meanwhile, the FLOPs increased by only 0.3 G, and the Parameters and Gradients increased by only 0.54 M. The FPS (Frames Per Second) test results indicate that the three optimization strategies had only a minor effect on the model FPS, with fluctuations remaining within 2 frames per second. After the overall optimization, the model FPS decreased by only 3.12, indicating a limited impact on detection speed. AE-YOLOv13-S improves the accuracy and recall of foreign object detection while maintaining computational efficiency and real-time performance. This provides strong technical support and practical value for the high-accuracy, stable, and non-destructive detection of foreign objects in Pu-erh ripe tea.

**Figure 9:**
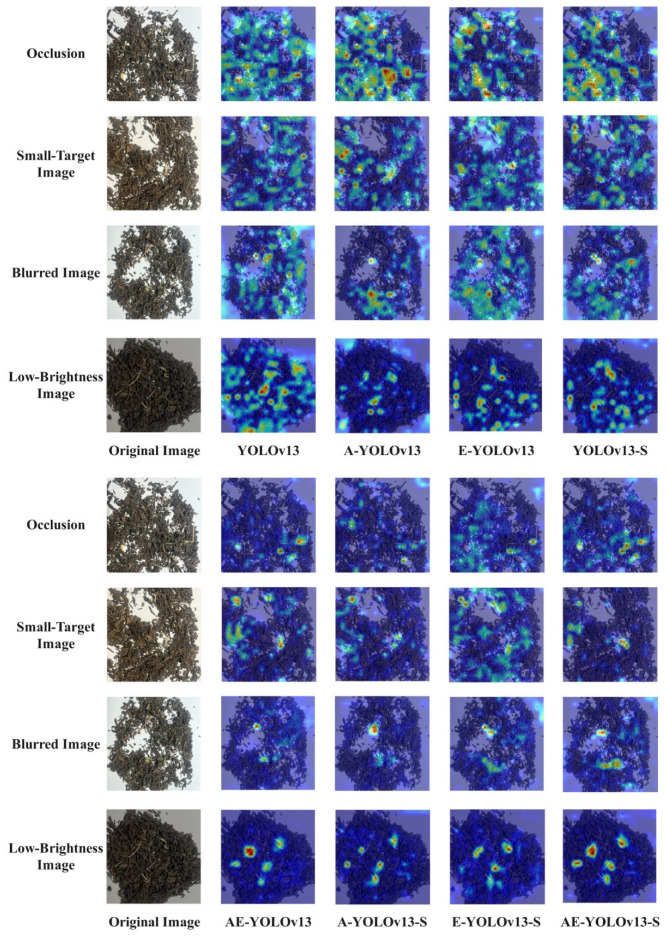
Grad-CAM heat map. The color intensity indicates the level of attention, with darker colors representing higher attention and lighter colors representing lower attention.

To further reveal the performance gains brought to YOLOv13 by optimization strategies such as Adaptive Sparse Self-Attention Network, Emulating Self-Attention with Convolution, and Scale-based Dynamic Loss, the model’s decision-making process was visually interpreted using Grad-CAM [32]. As a network visualization method based on gradient information, Grad-CAM obtains the contribution weight of each channel feature to the final discriminant result by calculating the gradient information of the target category relative to the specific convolutional layer feature map, and on this basis, the feature map is weighted and aggregated to generate the category activation heat map. This method can intuitively represent the spatial regions on which the model focuses during target recognition, thereby providing visual support for interpreting the basis of network discrimination and the mechanism of feature response. As shown in Figure 9, compared with YOLOv13, AE-YOLOv13-S exhibited a markedly stronger response to the representation of key foreign matter details under complex conditions such as occlusion, small targets, and blurred imaging, while the spatial focus on the target regions became more concentrated. The detection objects were manually selected based on the characteristics of common visual interference factors in the foreign object detection task for ripe Pu-erh tea, ensuring that the selected samples were highly representative.

### 3.4. Comparative Model Experiments

In the nondestructive foreign matter detection task for Pu-erh ripe tea, model detection performance directly determines recognition accuracy, operational stability, and engineering applicability in practical use. For a systematic analysis of overall model performance, the improved AE-YOLOv13-S under real detection conditions, this study selected seven object detection networks, namely AE-YOLOv13-S, YOLOv13, RT-DETR, SSD, YOLOv10, Faster RCNN, and RTMO, and carried out comparative experiments and performance tests on a unified dataset. As shown in Table 6, compared with YOLOv13, RT-DETR, SSD, YOLOv10, Faster RCNN, and RTMO, AE-YOLOv13-S improved Precision by 1.43, 4.17, 11.49, 2.33, 14.27, and 4.31 percentage points, respectively. Recall increased by 4.85, 7.67, 14.67, 6.78, 18.94, and 6.57 percentage points, respectively. F1 increased by 3.16, 5.94, 13.10, 4.59, 16.65, and 5.44 percentage points, respectively. mAP@50 increased by 2.69, 4.73, 12.06, 3.87, 16.80, and 4.34 percentage points, respectively. mAP@50:95 increased by 3.02, 4.91, 14.63, 4.24, 18.61, and 4.59 percentage points, respectively. The optimized AE-YOLOv13-S model showed good stability in recognizing all three types of foreign objects. It can accurately extract the structural features, particle morphology, and texture differences of foreign objects, achieving high detection accuracy and effectively meeting the requirements for recognizing and localizing multi-morphological foreign objects under complex Pu-erh ripe tea backgrounds.

**Figure 10:**
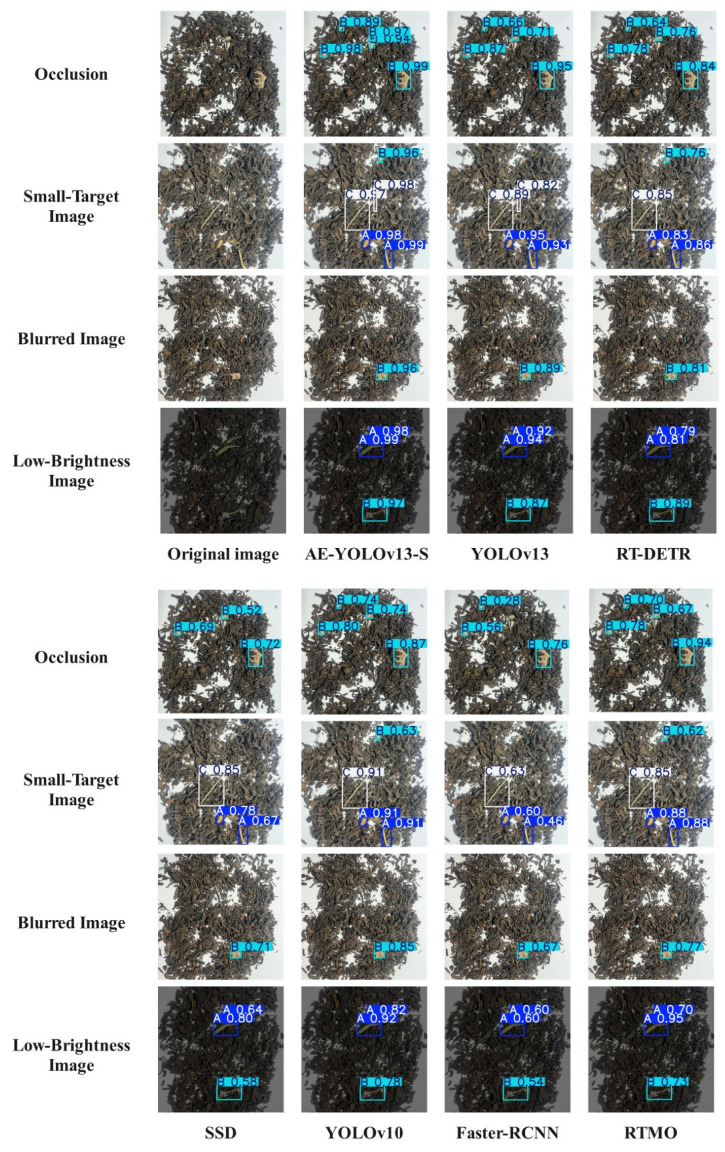
External validation. A represents bamboo leaf fragments, B represents small pebbles, and C represents small twigs.

To evaluate the robustness and scenario-specific adaptability of AE-YOLOv13-S in the nondestructive foreign matter detection task for Pu-erh ripe tea, this study conducted a comparative analysis of the detection performance of different models under typical complex conditions, including occlusion, low light, image blur, and small targets, and part of the results are shown in Figure 10. Here, A represents bamboo leaf fragments, B represents small pebbles, and C represents small twigs. To ensure the objectivity and credibility of the evaluation conclusions, external validation was carried out using independent samples, and all Pu-erh ripe tea samples were collected from Fengqing County, Lincang City, Yunnan Province [33]. The image acquisition procedure for the external validation samples was kept consistent with that used for the data employed in model training. A total of 100 foreign object images were collected to evaluate the detection performance of the model on external samples. The experimental results showed that, compared with other reference models, AE-YOLOv13-S achieved clearly higher confidence under complex conditions, and the phenomenon of missed detection was markedly alleviated. The improved AE-YOLOv13-S network demonstrated stronger robust detection capability and task adaptability in complex natural scenes, thereby providing reliable theoretical support and technical evidence for the nondestructive foreign matter detection of Pu-erh ripe tea.

## 4. Conclusions and Discussion

To address the loss of fine details, the weakening of discriminative features, and the frequent occurrence of missed and false detections caused by strong texture interference, small scale foreign matter, significant local occlusion, and severe confusion from pseudo foreign matter in the natural spreading and withering scenario of Pu-erh ripe tea, this study drew on the visual mechanisms of primates and proposed a deep learning network, AE-YOLOv13-S, for nondestructive foreign matter detection in Pu-erh ripe tea. YOLOv13 was used as the baseline network, and the Adaptive Sparse Self-Attention Network was adopted to optimize the backbone of the network by drawing on the biologically inspired sequential cognitive characteristics of primates, including target search, local verification, selective integration, and final decision making. Drawing on the hierarchical information processing mechanism of primates from peripheral perception to central fine analysis, part of the Conv modules in the network were optimized through Emulating Self-Attention with Convolution. Inspired by the visual perception mechanism of primates, Scale-based Dynamic Loss was introduced to optimize the loss function of the network.

The results show that, compared with the original YOLOv13, AE-YOLOv13-S reduced Box Loss, Cls Loss, and DFL Loss during the training stage by 6.76%, 6.52%, and 8.65%, respectively. During the validation stage, the three types of loss decreased by 6.58%, 16.39%, and 8.33%, respectively. The multi-module collaborative optimization strategy adopted in this study effectively enhances the model’s ability to construct discriminative features. In addition, compared with the baseline model, AE-YOLOv13-S improved Precision, Recall, mAP@50, and mAP@50:95 by 1.43, 4.85, 2.69, and 3.02 percentage points, respectively, while the FLOPs increased by only 0.3 G. These results indicate that the improved model achieves systematic improvements in detection performance while maintaining a low computational cost. Compared with mainstream detection models such as YOLOv13, RT-DETR, SSD, YOLOv10, Faster RCNN, and RTMO, AE-YOLOv13-S achieved the best overall detection performance, demonstrating stronger robustness and task adaptability, and effectively reducing the risk of missed and false detections.

To some extent, this study expands the application scope of deep learning and bio-inspired vision theory in the field of nondestructive detection of agricultural products. It not only provides a new technical pathway for foreign matter detection during the processing of Pu-erh ripe tea, but also offers a practically meaningful technical solution for intelligent quality control and food safety assurance in tea processing. The results indicate that drawing on mechanisms such as attentional resource allocation, candidate region screening, hierarchical information processing, and scale-adaptive perception in primate vision can effectively improve the robustness and generalization ability of the model under complex background conditions.

Nevertheless, this study still has room for further extension. The current dataset remains relatively limited in scale, and the foreign matter categories mainly include bamboo leaf fragments, small twigs, and small pebbles. Although these categories can to some extent represent the typical distribution of foreign matter during the processing of Pu-erh ripe tea, they still provide insufficient coverage of the full range of foreign matter types encountered in actual production. In addition, the sample size of the test set in this study remains relatively small, making it difficult to fully cover all variations caused by different batches of ripe Pu-erh tea, different foreign object morphologies, different illumination conditions, and complex interference factors in real production lines. The current study analyzed the model results only based on visual recognition metrics, and statistical methods have not yet been introduced to compare model performance.

In future work, our team will further expand the data scale under real production scenarios and combine repeated experiments, cross-validation, and statistical variance analysis to more systematically evaluate the generalization ability and stability of the model. Future research will also conduct online detection experiments using actual tea sorting equipment to further verify the model’s real-time processing capability, deployment feasibility, and long-term operational stability in industrial scenarios. The present optimization mainly achieves a structural incorporation of mechanisms in primate vision, including selective attention, hierarchical processing, and scale regulation. However, the modeling of higher-level biological visual behaviors, such as dynamic scanning, temporal attention transfer, and multimodal collaborative perception, remains insufficient. Future research will further improve the algorithm performance and extend the proposed method to nondestructive detection scenarios involving other tea types and agricultural products, such as green tea, black tea, oolong tea, white tea, grains, and nuts, where food detection tasks involve complex textured backgrounds and small foreign object targets. This will provide technical reference and methodological support for intelligent sorting, quality and safety inspection, and production process automation of tea and agricultural products.

## Figures and Tables

**Table 1:** Parameters of the improved YOLOv13 network.

ID	From	Params	Module	Arguments
0	−1	464	Conv	[3, 16, 3, 2]
1	−1	2368	Conv	[16, 32, 3, 2, 1, 2]
2	−1	5792	DS-C3k2	[32, 64, 1, False, 0.25]
3	−1	9344	Conv	[64, 64, 3, 2, 1, 4]
4	−1	20,800	DS-C3k2	[64, 128, 1, False, 0.25]
5	−1	17,792	DSConv	[128, 128, 3, 2]
6	−1	174,720	A2C2f	[128, 128, 2, True, 4]
7	−1	34,432	DSConv	[128, 128, 3, 2]
8	−1	677,120	A2C2f	[256, 256, 2, True, 1]
9	−1	238,336	ASSANet (Optimized Modules)	[256, 256]
10	[4, 6, 8]	273,536	HyperACE	[128, 128, 1, 4, True, True, 0.5, 1, ‘both’]
11	−1	0	Upsample	[None, 2, ‘nearest’]
12	10	33,280	DownsampleConv	[128]
13	[6, 10]	1	FullPAD Tunnel	[]
14	[4, 11]	1	FullPAD Tunnel	[]
15	[9, 12]	1	FullPAD Tunnel	[]
16	−1	0	Upsample	[None, 2, ‘nearest’]
17	[−1, 13]	0	Concat	[1]
18	−1	165,024	C3k2-ESC (Optimized Modules)	[384, 128, 1, True]
19	[−1, 10]	1	FullPAD Tunnel	[]
20	18	0	Upsample	[None, 2, ‘nearest’]
21	[−1, 14]	0	Concat	[1]
22	−1	51,000	C3k2-ESC (Optimized Modules)	[256, 64, 1, True]
23	11	8320	Conv	[128, 64, 1, 1]
24	[22, 23]	1	FullPAD Tunnel	[]
25	−1	36,992	Conv	[64, 64, 3, 2]
26	[−1, 19]	0	Concat	[1]
27	−1	140,448	C3k2-ESC (Optimized Modules)	[192, 128, 1, True]
28	[−1, 10]	1	FullPAD Tunnel	[]
29	27	147,712	Conv	[128, 128, 3, 2]
30	[−1, 15]	0	Concat	[1]
31	−1	533,936	C3k2-ESC (Optimized Modules)	[384, 256, 1, True]
32	[−1, 12]	1	FullPAD Tunnel	[]
33	[24, 28, 32]	431,452	Detect	[3, [64, 128, 256]]

**Table 2:** Software and hardware parameters.

Software and Hardware Names	Configuration Parameters
Operating System	Windows 10
Processor	12th Gen Intel(R) Core(TM)i5-12600KF
Graphics Card	NVIDIA GeForce RTX 4060 Ti (16 GB)
Solid-State Drive	Kingston NV2 1TB PCIe 4.0 NVMe M.2
Memory	Colorful 32 (16 × 2) G 3200 DDR4
Driver	NVIDIA-SMI 561.09
CUDA	CUDA Version: 12.6
Programming Language	Python 3.9
Network Development	PyCharm 2024

**Table 3:** Parameter settings.

Parameter	Value
SGD Momentum	0.937
Optimizer Weight Decay	0.001
Epochs	500
Batch	16
Input image size	640 × 640
Initial learning rate	0.1
Box loss gain	7.5
Classification loss gain	0.5
Distribution Focal Loss gain	1.5

**Table 4:** Comparative experiment on data augmentation.

Model	Without Data Augmentation				With Data Augmentation			
	Precision (%)	Recall (%)	mAP@50 (%)	mAP@50:95 (%)	Precision (%)	Recall (%)	mAP@50 (%)	mAP@50:95 (%)
AE-YOLOv13-S	90.26	91.23	92.64	82.84	92.62	93.34	94.91	85.67
YOLOv13	88.30	85.94	89.61	79.48	91.19	88.49	92.22	82.65
RT-DETR	84.87	83.66	87.38	77.31	88.45	85.67	90.18	80.76
SSD	78.89	75.43	79.97	67.42	81.13	78.67	82.85	71.04
YOLOv10	87.04	83.95	88.24	78.49	90.29	86.56	91.04	81.43
Faster-RCNN	75.76	71.93	75.62	63.78	78.35	74.40	78.11	67.06
RTMO	85.41	84.19	87.98	77.53	88.31	86.59	90.57	81.08

**Table 5:** Results of the ablation study.

Model	Precision (%)	Recall (%)	mAP@50 (%)	mAP@50:95 (%)	FLOPs (G)	Parameters	FPS
YOLOv13	91.19	88.49	92.22	82.65	6.4	2,460,496	64.10
A-YOLOv13	91.63	91.47	93.38	84.09	6.6	2,698,832	62.11
E-YOLOv13	91.58	91.08	93.16	83.32	6.5	2,764,344	63.29
YOLOv13-S	91.85	90.65	92.87	83.16	6.4	2,460,496	64.52
AE-YOLOv13	92.38	91.76	94.09	85.12	6.7	3,002,680	60.61
A-YOLOv13-S	92.31	91.66	93.78	84.73	6.6	2,698,832	61.73
E-YOLOv13-S	92.17	91.31	93.58	84.64	6.5	2,764,344	62.89
AE-YOLOv13-S	92.62	93.34	94.91	85.67	6.7	3,002,680	60.98

**Table 6:** Results of comparative model experiments.

Model	Precision (%)	Recall (%)	F1 (%)	AP1@50 (%)	AP2@50 (%)	AP3@50 (%)	mAP@50 (%)	mAP@50:95 (%)
AE-YOLOv13-S	92.62	93.34	92.98	96.54	96.98	91.21	94.91	85.67
YOLOv13	91.19	88.49	89.82	94.28	95.15	87.23	92.22	82.65
RT-DETR	88.45	85.67	87.04	92.05	92.07	86.42	90.18	80.76
SSD	81.13	78.67	79.88	84.76	84.82	78.97	82.85	71.04
YOLOv10	90.29	86.56	88.39	92.73	93.83	86.56	91.04	81.43
Faster-RCNN	78.35	74.40	76.33	80.03	80.08	74.22	78.11	67.06
RTMO	88.31	86.59	87.44	92.42	92.45	86.84	90.57	81.08

Note: AP1 denotes the AP value for bamboo leaf fragments, AP2 denotes the AP value for small pebbles, and AP3 denotes the AP value for small twigs.

## Data Availability

The original code presented in the study is openly available in IEEE DataPort at https://dx.doi.org/10.21227/33zg-dq58 (accessed on 27 April 2026).

## References

[1] Zheng Y., Liu Y., Han S., He Y., Liu R., Zhou P. (2024). Comprehensive evaluation of quality and bioactivity of kombucha from six major tea types in China. Int. J. Gastron. Food Sci..

[2] Kuang Z., Yu X., Guo Y., Cai Y., Hong W. (2024). Design of a Multimodal Detection System Tested on Tea Impurity Detection. Remote Sens..

[3] Li X., Sanaeifar A., Zhang S., Zhan Z., He Y. (2024). Recent Technological Advances in Tea Quality and Safety. Natural Products in Beverages: Botany, Phytochemistry, Pharmacology and Processing.

[4] Wang Y., Zhang C., Wang Z., Liu M., Zhou D., Li J. (2024). Application of lightweight YOLOv5 for walnut kernel grade classification and endogenous foreign body detection. J. Food Compos. Anal..

[5] Yu H., Ma M., Zhang B., Wang A., Zhong G., Zhou Z., Liu C., Li C., Fang J., He Y. (2025). Bionic Sensors for Biometric Acquisition and Monitoring: Challenges and Opportunities. Sensors.

[6] Ding Z., Wang M., Hu B., Chen Z., Dong C. (2025). Impurity detection of premium green tea based on improved lightweight deep learning model. Food Res. Int..

[7] Huang S., Lin C., Jiang X., Qu Z. Brstd: Bio-inspired remote sensing tiny object detection. Proceedings of the IEEE Transactions on Geoscience and Remote Sensing.

[8] Wang Z., Zhang S., Chen Y., Xia Y., Wang H., Jin R., Wang C., Fan Z., Wang Y., Wang B. (2025). Detection of small foreign objects in Pu-erh sun-dried green tea: An enhanced YOLOv8 neural network model based on deep learning. Food Control.

[9] Ye X., He M., Wang J., Huang L., Xu J., Zhang R., Li B. (2026). An Approach to Crayfish Weight Estimation Based on Pose Awareness. Appl. Sci..

[10] Yang T., Lin X., Xu Y., Liu Z., Luo Z. (2025). Potential risks in tea and emerging monitoring technologies: A review. Agric. Prod. Process. Storage.

[11] Lei M., Li S., Wu Y., Hu H., Zhou Y., Zheng X., Ding G., Du S., Wu Z., Gao Y. (2025). Yolov13: Real-time object detection with hypergraph-enhanced adaptive visual perception. arXiv.

[12] Pan J., Sun L., Song L., Dong J., Yang J., Zhao M. (2026). Adaptive Sparse Self-Attention for Efficient Image Super-resolution and beyond. IEEE Trans. Pattern Anal. Mach. Intell..

[13] Lee D., Yun S., Ro Y. Emulating self-attention with convolution for efficient image super-resolution. Proceedings of the IEEE/CVF International Conference on Computer Vision (ICCV).

[14] Yang J., Liu S., Wu J., Su X., Hai N., Huang X. (2025). Pinwheel-shaped convolution and scale-based dynamic loss for infrared small target detection. Proc. AAAI Conf. Artif. Intell..

[15] Huang Q., Kuang Y., Zhou H., Li X., Yin L. (2024). Biodiversity Conservation in Xishuangbanna, China: Diversity Analysis of Traditional Knowledge Related to Biodiversity and Conservation Progress and Achievement Evaluation. Diversity.

[16] Zenkl R., McDonald B.A., Walter A., Anderegg J. (2025). Towards high throughput in-field detection and quantification of wheat foliar diseases using deep learning. Comput. Electron. Agric..

[17] Ye X., Qin X., Zhan L., Wang J., Chen Y. (2025). Research on a Fusion Technique of YOLOv8-URE-Based 2D Vision and Point Cloud for Robotic Grasping in Stacked Scenarios. Appl. Sci..

[18] Zhang J., Su H., Zhang T., Tian H., Fan B. (2025). Multi-Scale Fusion Underwater Image Enhancement Based on HSV Color Space Equalization. Sensors.

[19] Jang H., Tong F. (2024). Improved modeling of human vision by incorporating robustness to blur in convolutional neural networks. Nat. Commun..

[20] Fan Y., Wang Y., Liang D., Chen Y., Xie H., Wang F. (2024). Low-FaceNet: Face recognition-driven low-light image enhancement. IEEE Trans. Instrum. Meas..

[21] Guo X., Yang C., Wang Z., Zhang J., Zhang S., Wang B. (2025). Research on the Yunnan Large-Leaf Tea Tree Disease Detection Model Based on the Improved YOLOv10 Network and UAV Remote Sensing. Appl. Sci..

[22] Ye X., Huang L., Wang J., Hu X., Xu J., Zhang C. (2026). A Lightweight PCB Defect Detection Algorithm Based on an Improved YOLOv11s. IEEE Access.

[23] Sanjalawe Y., Makhadmeh S.N., Almiani M., Mari S.A. (2025). HyperFallNet: Human fall detection in real-time using YOLOv13 with hypergraph-enhanced correlation learning. IEEE Access.

[24] Kar K., DiCarlo J.J. (2024). The quest for an integrated set of neural mechanisms underlying object recognition in primates. Annu. Rev. Vis. Sci..

[25] AliAkbarpour H., Moori A., Khorramdel J., Blasch E., Tahri O. (2024). Emerging trends and applications of neuromorphic dynamic vision sensors: A survey. IEEE Sens. Rev..

[26] Xu J., Girardi-Schappo M., Beique J.C., Longtin A., Maler L. (2024). Shortcutting from self-motion signals reveals a cognitive map in mice. eLife.

[27] Zhang S., Guo X., Tan M., Yang C., Wang Z., Li G., Wang B. (2025). DE-YOLOv13-S: Research on a Biomimetic Vision-Based Model for Yield Detection of Yunnan Large-Leaf Tea Trees. Biomimetics.

[28] Zhong H., Zhang Y., Shi Z., Zhang Y., Zhao L. (2025). PS-YOLO: A Lighter and Faster Network for UAV Object Detection. Remote Sens..

[29] Yang Y., Chen S., Li J. (2026). The Study of Improved YOLOv13-Based Method for Detection of Industrial Surface Defects. Symmetry.

[30] Terven J., Cordova-Esparza D.M., Romero-González J.A., Ramírez-Pedraza A., Chávez-Urbiola E.A. (2025). A comprehensive survey of loss functions and metrics in deep learning. Artif. Intell. Rev..

[31] Mao M., Lee A., Hong M. (2024). Efficient Fabric Classification and Object Detection Using YOLOv10. Electronics.

[32] Ennab M., Mcheick H. (2025). Advancing AI Interpretability in Medical Imaging: A Comparative Analysis of Pixel-Level Interpretability and Grad-CAM Models. Mach. Learn. Knowl. Extr..

[33] Chai R., Tian N., Wan G., Liu S., Zhan J., Li X., Bian H., Gao C., Xia X., Wang D. (2025). Automated detection of early-stage osteonecrosis of the femoral head in adult using YOLOv10: Multi-institutional validation. Eur. J. Radiol..

